# Assessing Level of Knowledge and Uptake of Hepatitis B Vaccination among Health Care Workers at Juba Teaching Hospital, Juba City, South Sudan

**DOI:** 10.1155/2020/8888409

**Published:** 2020-12-24

**Authors:** John Bosco Alege, Godfrey Gulom, Alphonse Ochom, Viola Emmanuel Kaku

**Affiliations:** ^1^Institute of Public Health and Management, Clarke International University, Kampala, Uganda; ^2^School of Public Health and Applied Human Sciences, Kenyatta University, Nairobi, Kenya

## Abstract

**Background:**

Chronic hepatitis B (CHB) virus (HBV) infection remains a severe problem worldwide. An estimated 240–400 million persons are reported to have chronic HBV infection, and the annual mortality from HBV-related complications including cirrhosis and hepatocellular carcinoma was 600,000 persons. In Sub-Saharan Africa, the prevalence of HBV chronic infection is particularly high while in South Sudan, hepatitis B remains a serious problem of public health importance with health care workers being more at greater risk. Vaccination coverage against HBV is low among all age groups, yet vaccination status among health care workers is not even known in South Sudan. This study aimed at assessing viral hepatitis B vaccination uptake among health care workers at Juba Teaching Hospital, Juba City, South Sudan.

**Objective:**

To assess the uptake of viral hepatitis B vaccination among health care workers in Juba Teaching Hospital, Juba City, South Sudan.

**Methods:**

An analytical cross-sectional study design was conducted targeting 154 health workers. A convenient sampling procedure was used to recruit study participants. Questionnaires were used to collect data. SPSS version 20.0 was used for data analysis. Chi-square tests were used to determine the association between the uptake of hepatitis B vaccination and individual and health facility factors. Multivariable analysis was conducted. Adjusted OR was used to interpret the findings.

**Results:**

Uptake of hepatitis B vaccination was found to be low at 44.20%, only 48.8% had received one dose, 29.1% received two doses, and 22.1% had received all three doses. Being married (*p* ≤ 0.008), knowing that hepatitis B can be prevented by vaccination (*p* ≤ 0.001), knowing that HBV can be got through unprotected sexual intercourse (*p* ≤ 0.001), awareness of where to get hepatitis B vaccination from (*p* ≤ 0.001), availability of vaccines in the health facility (*p* ≤ 0.027), and availability of guidelines followed by all health workers in this facility (*p* ≤ 0.006) were the factors independently associated with the uptake of hepatitis B vaccination.

**Conclusion:**

The uptake of hepatitis B vaccination among health workers at Juba Teaching Hospital was low (22.1%), putting health workers at great risk of HBV infection. Having knowledge about hepatitis B vaccination and unprotected sexual intercourse were individual factors associated with hepatitis B vaccination. Availability of the vaccine and vaccination guidelines were the health-related factors associated with hepatitis B vaccination. The government of South Sudan through the Ministry of Health should first track approval of the viral hepatitis B vaccination policy and ensure that it is adopted and implemented by all hospitals. Health care workers must be prioritized and mandatorily vaccinated against viral hepatitis B.

## 1. Background to the Study

Hepatitis B infection is a worldwide public health problem. Hepatitis B virus infection remains a severe problem worldwide but more prevalent in Sub-Saharan Africa with 240–400 million persons reported to have chronic HBV infection by Roberts H. et al., [1] only 9% of persons living with hepatitis B have tested and are aware of their status [2]. Without the scale-up of existing interventions, there will be a cumulative 63 million new cases of chronic infections and HBV-related deaths between 2015 and 2030 [3]. Health care workers are a high-risk group in terms of exposure to HBV; thus, the observance of the universal safety precautions and vaccination of people at risk is vital. A study in Kenya found that 80% of health care workers were vaccinated against HBV, and out of those, only 48% had received all three doses [4]. Another study conducted in Tanzania on hepatitis B vaccination coverage among health care workers at a national hospital by [5] established that out of 348 HCWs, 198 (56.9%) had received at least one dose of hepatitis B vaccination, while only 117 (33.6%) were fully vaccinated, a slightly higher coverage compared to South Sudan. However, the prevalence of HBV among the general population is estimated to be 22% in South Sudan, Schweitzer et al., [6]. There is no national data on the prevalence of HBV among HCWs, including data on the proportion of those who are fully vaccinated against HBV.

Thus, if the above HBV problem is not fully contained, the country will experience an increase in HBV incidence, prevalence, high morbidity, and mortality, because HCWs are a vulnerable population and will mediate transmission. It is against this background that this study was conducted.

## 2. Methods

An analytical cross-sectional study design was used to assess the uptake of hepatitis B vaccination among health care workers at Juba Teaching Hospital, South Sudan. Juba Teaching Hospital (study site) is the national referral hospital that provides super specialized services and receives referrals from all over the ten states or regions of South Sudan. It is a well-staffed hospital hosting specialties of varied disciplines rendering health care services for the population in South Sudan. A total of 154 study participants were recruited to participate in the study. The sample size was determined using Yamane finite sample size determination formula. Juba Teaching Hospital was purposively selected for this study because it is a national referral and specialized hospital with several services offered. It employs the largest number of health workers who met the inclusion criteria in this study. This study utilized a convenience sampling technique to select and interview the study participants at the study site. All health workers in Juba Teaching Hospital aged 18–59 years involved in handling patients in different departments, such as laboratory, emergency care unit, in-patient department, theater, and labour suite, and had worked in Juba Teaching Hospital for a period of more than six months were recruited into this study. A precoded self-administered semistructured questionnaire was used to collect data on the vaccination status of health care workers in Juba Teaching Hospital. Options of “Yes” for vaccinated and “No” for nonvaccinated health care workers, based on self-reporting, were used to determine the vaccination status of health care workers.

The questionnaires were written in English, and a translated copy in the Arabic language was made available for health care workers who studied in Arabic. *Data Analysis*. The sociodemographic variables and individual and health facility factors were analyzed using Statistical Package for Social Sciences (SPSS) version 20.0, and the descriptive data was analyzed using Chi-square tests to assess the association between uptake of hepatitis B vaccination, sociodemographic factors, and individual and health facility factors. Descriptive data was analyzed at three different levels; at the univariate level, data was presented using tables of frequencies, percentages, and a pie chart. At the bivariate level, the data produced was used to compare the health workers' status of HBV vaccination uptake. The potential predictors of HBV vaccination uptake were analyzed, and a *p* value less than 0.05 at CI of 95% was considered statistically significant.

Multivariable analysis was conducted for factors that were found to be significantly associated with a dependent variable at the bivariate level. The logistic regression model was fitted to further determine the strength of association between independent and dependent variables, and Adjusted OR was used to interpret the results. The analysis was stratified to cadre since the exposure and vaccination uptake varies between cadres. *p* value less than 0.05 was considered significant.

Ethical approval to conduct this study was obtained from the Clarke International University Research and Ethics Committee (REC). Thereafter, permission to collect data was sought from the Juba Teaching Hospital research ethics committee together with the hospital/medical superintendent. Consent was obtained from the respondents; confidentiality of data collected was ensured to protect the privacy of the study participants. There was the utmost respect for all study participants during data collection.

## 3. Results

A total of 154 respondents participated in the study yielding a 100% response rate. The majority (69/154 (44.8%)) of the respondents were in the age bracket of 18–30 years, 92/154 (59.7%) were male, 80/154 (51.9%) were married.

### 3.1. Level of Awareness

A structured questionnaire was used to assess the level of awareness of study respondents about hepatitis B infection. The majority of the respondents were aware that hepatitis B is a viral infection and that it can be acquired through body fluids (80.5%), needle sticks (77.3%), and if vaccination is effective in preventing hepatitis B infection (79.2%), and only a few of respondents (20.1%) knew that receiving a full dose of HBV was vital in preventing one from HBV infections.

### 3.2. Hepatitis B Vaccination Uptake by Study Respondents

The results show that only 44.2% of the study participants had been vaccinated against viral hepatitis B infection, while more than half of the respondents (55.8%) had never been vaccinated against hepatitis B. However, out of the total 154 study participants, only 48.8% had received one dose, 29.1% got two doses, and only 22.1% had received all the three recommended doses of the vaccine. The reasons for not completing the vaccine doses were busy schedules (63%), forgetting vaccination appointments (53.9), and long waiting time for the next appointment date (52.6), while 36.4% felt they were already protected, so no need to complete the 3 doses. The main reasons for nonvaccination as stated by respondents were lack of health education (64.9%), high cost of HBV vaccines (55.2%), lack of free vaccination opportunities (50.0%), busy schedules (49.4%), lack of the vaccine (46.10%), and following standard precautions (47.40%).

### 3.3. Individual Factors Associated with Hepatitis B Vaccination among Health Care Workers in Juba Teaching Hospital

Table 1 shows the individual factors associated with hepatitis B vaccination; respondents who felt that they are at low risk of acquiring HBV (*χ*^2^ = 21.006, *p* ≤ 0.001), lack of willingness to secure time to go for HBV vaccination (*χ*^2^ = 18.545, *p* ≤ 0.001), hepatitis B infection that can be prevented by vaccination (*χ*^2^ = 4.210, *p* ≤ 0.031), getting HBV through unprotected sexual intercourse (*χ*^2^ = 22.990, *p* ≤ 0.006), awareness of where to get hepatitis B vaccination (*χ*^2^ = 4.155, *p* ≤ 0.011), poor management of infectious medical waste that predisposes me to HBV infection (*χ*^2^ = 7.035, *p* ≤ 0.030), all HCWs that are at high risk of HBV infection (*χ*^2^ = 6.054, *p* ≤ 0.048), feeling of being susceptible to HBV infection (*χ*^2^ = 7.014, *p* ≤ 0.030), willingness to receive HBV vaccination ones offered a chance (*χ*^2^ = 14.109, *p* ≤ 0.028), and willingness to manage hospital infectious waste properly (*χ*^2^ = 19.105, *p* ≤ 0.011) were all significantly associated with hepatitis B vaccination uptake.

### 3.4. Health Facility Factors Associated with Hepatitis B Vaccination

Table 2 shows the health facility factors that were associated with hepatitis B vaccination, and they included availability of HBV vaccines (*χ*^2^ = 9.640, *p* ≤ 0.023), affordability of vaccines (*χ*^2^ = 15.064, *p* ≤ 0.004), frequency of use of PPE (*χ*^2^ = 15.333, *p* ≤ 0.001), Information Education and Communication (IEC) materials being available (*χ*^2^ = 9.662, *p* ≤ 0.022), availability of HBV vaccination guidelines (*χ*^2^ = 5.496, *p* ≤ 0.014), and knowledge of where to get HBV vaccination from (*χ*^2^ = 10.082, *p* ≤ 0.040).

### 3.5. Multivariable Analysis of Factors Associated with Hepatitis B Vaccination among Health Care Workers in Juba Teaching Hospital

Table 3 shows the multivariable analysis of factors associated with hepatitis B vaccination among the study participants. Study participants who said that the health facility has vaccines were 3.258 times more likely to get hepatitis B vaccination than those who said the health facility does not have HBV vaccines (Adjusted OR = 3.258, 95% CI 1.418–6.497, *p* ≤ 0.027). Respondents who were married were 8.942 times more likely to have been vaccinated against HBV than those who were not married (Adjusted OR = 8.942, 95% CI 4.313–14.849, *p* ≤ 0.008). Respondents who knew that hepatitis B infection can be prevented by vaccination were 4.192 times more likely to get HBV vaccination compared to those who did not know (Adjusted OR = 4.192, 95% CI 2.395–9.162, *p* ≤ 0.001). Respondents who knew that one can get HBV through unprotected sexual intercourse were 2.135 more likely to get compared to those who did not know that one can get HBV through unprotected sexual intercourse (Adjusted OR = 2.135, 95% CI 1.159–5.371, *p* ≤ 0.001). Respondents who disagreed that they were aware of where to get hepatitis B vaccination were less likely to get HBV vaccination compared to those who agreed that they were aware of where to get hepatitis B vaccination from (Adjusted OR = 0.177, 95% CI 0.081–0.475, *p* ≤ 0.001). Respondents who disagreed that they were willing to receive HBV vaccination once offered chance were less likely to get HBV vaccination compared to those who were willing to receive HBV vaccination once offered chance (Adjusted OR = 0.698, 95% CI 0.478–0.944, *p* ≤ 0.010). Respondents who agreed that they were willing to receive HBV vaccination once offered chance were less likely to get HBV vaccination compared to those who were not sure about receiving HBV vaccination once offered chance (Adjusted OR = 9.392, 95% CI 6.016–12.165, *p* ≤ 0.004). Respondents who reported the availability of guidelines followed by health workers in the facility were 6.228 times more likely to get HBV vaccination compared to those who said there were no guidelines followed by health workers in the health facility (Adjusted OR = 6.228, 95% CI 3.151–8.902, *p* ≤ 0.006).

Respondents who had knowledge of where to get HBV vaccination from were 7.042 times more likely to get HBV vaccination than those who had no knowledge of where to get HBV vaccination from (Adjusted OR = 7.042, 95% CI 4.482–11.196, *p* ≤ 0.001).

## 4. Discussion

The uptake of hepatitis B vaccination among health workers at Juba Teaching Hospital is low, implying that health workers in South Sudan are at greater risk of acquiring hepatitis infections.

The lack of health education, high cost of HBV vaccine, lack of free vaccination opportunities, lack of the vaccine, and following standard precautions are the main factors for low uptake of hepatitis B vaccination.

### 4.1. Uptake of Hepatitis B Vaccination

This study found that the level of uptake of hepatitis B vaccination among the health workers was low at 44.2%. This could be attributed to the lack of approved policy by the Ministry of Health on hepatitis B vaccination, especially targeting health care workers who are highly vulnerable in South Sudan. This is not consistent with findings from a study conducted in Zambia by [7], in which it was established that 64 (19.3%) of the health care workers were vaccinated against hepatitis B, with 35 (54.7%) of these being fully vaccinated and 29 (45.3%) being partially vaccinated against HBV. In addition, other studies conducted in the East and Horn of Africa show a higher uptake of HBV vaccination among health workers; for instance, a study conducted in Tanzania [5] established that out of 348 HCWs, 198 (56.9%) were vaccinated, while in Ethiopia, out of 286 HWs (study respondents) [8] found that 58.6% received the full dose (3 doses) of the vaccine. First, targeted and compulsory vaccination of HCWs in South Sudan has not been prioritized. Second, MoH in South Sudan, for a long time now, has been focusing on emergency health programming given the fragile nature of the country due to insecurity and armed conflict. The slightly above half of the HCWs vaccinated accessed the services from the private sector and they had to pay out of pocket.

In this study, only 22.1% of the respondents had received all three doses of the HBV vaccines while 29.1% of the respondents had got two doses of the vaccines and 48.8% had received only one dose. This is probably because the majority of the HCWs have just started to get the vaccination because of the comprehensive sensitization among the health workers. The proportion of study participants who completed all the three doses in this study is slightly higher than the finding from an institutional-based cross-sectional study in Ethiopia conducted among 423 respondents [9] who noted that 16.1% of the respondents had ever received HBV vaccination and 12.9% of the health workers had received all the three doses of HBV. Mbanga et al. [10], in another study conducted in Cameroon that recruited 714 respondents, also found that 6.81% had attained complete vaccination against HBV.

This implies that generally completing the mandatory three doses of HBV vaccination is a challenge among HCWs in Sub-Saharan Africa.

This study found that 64.9% of the respondents were not vaccinated because they never receive health education, 55.2% were already infected, 50.0% never had a chance, and 49.4% had no time to go for vaccination. All the above factors that acted as barriers to the uptake of HBV vaccine by HCWs could mainly be due to the lack of guidelines and policy on HBV vaccination in the country. The findings are similar to findings from another study carried out among 286 HCWs [8] in Ethiopia in which it was reported that the reasons for not being vaccinated were related to unavailability of vaccines (58.2%), high cost of vaccines (18.5%), being busy with some other courses (32.8%) which was one of the reasons for not receiving a full course of the vaccine. Similarly, a cross-section study conducted in South Africa among 400 respondents [11] also found that 55.7% of HCWs had received at least one dose of HBV vaccine, and 53 (15.4%) were fully vaccinated having received all the three doses of HB vaccine as recommended by the World Health Organization (WHO). Mbanga et al. [10] conducted a cross-sectional study in Cameroon among 714 respondents; they established that poor HBV vaccination uptake was attributed to worries and fear of side effects, as well as fear of being infected by the vaccine. This study among HCWs in South Sudan found that slightly more than half of the respondents accepted that they were at risk of HBV infection. This is probably because HCWs are a vulnerable population. After all, they work in the clinical areas managing patients who may not be knowing their HBV status and could therefore be exposed to HBV infection.

This is in line with findings from a survey conducted in Nigeria among 341 respondents [12] who found that 55.3% of the respondents rated themselves at high risk of occupational exposure to HBV infection, 22.4% did not know their risk of exposure, 5.3% considered themselves at no risk of exposure to HBV infection, whereas 5.9% and 38 11.2% perceived low risk and moderately at risk, respectively. Similarly, a hospital-based cross-sectional study conducted in Ethiopia [13] also found that unprotected mucocutaneous fluid contact on intact skin (66.7%), sharp needle injury (39.6%), and body fluid splashes (28.1%) were among few reported forms of exposure which increased perception levels among health care workers.

### 4.2. Individual Factors Associated with Hepatitis B Vaccination Uptake

In this study, 44.8% of the respondents were in the age bracket of 18–30 years, and the age of the respondents was not significantly associated with hepatitis B vaccination. This is probably because all HCWs, irrespective of age, are required to be vaccinated according to the WHO guidelines. The findings are inconsistent with a study conducted in Nigeria among 288 primary health care workers [14] who found that the mean age of respondents who were vaccinated was 40 ± 4.8 years, and the practice of vaccination was found to increase with the age of respondents and this was statistically significant (*p* ≤ 0.001). The variance in the study findings could probably arise from the difference in the study settings and study objective. This study found that 59.7% of the respondents were male and the sex of the health workers was not significantly associated with hepatitis B vaccination. This is probably because both male and female HCWs were equally at high risk and exposed to HBV infection; hence, being a male or female HCW did not matter. This is in disagreement with a study conducted in Thailand among 280 respondents [15]; it found a significant difference in attitude towards hepatitis B vaccination between males and females.

In this study, 51.9% of the respondents were married. Marital status was significantly associated with hepatitis B vaccination; respondents who were married were 8.942 times more likely to have been vaccinated compared to those who were not married. This is probably because the health workers knew that HBV can be acquired through sexual intercourse; hence, to protect against infection, one needed to get vaccinated. Nevertheless, this is not in line with a hospital-based study conducted in Nigeria among 209 respondents [16]; the findings noted that the marital status of the respondents did not have any significant relationship with HBV vaccination uptake. HCWs were less likely to receive vaccination whether married or not.

This study established that 79.2% of the respondents said hepatitis B infection can be prevented by vaccination. The knowledge that hepatitis B can be prevented by vaccination was significantly associated with hepatitis B vaccination. Respondents who knew that hepatitis infection can be prevented by vaccination were 4.192 times more likely to get HBV vaccination compared to those who did not know (Adjusted OR = 4.192, 95% CI 2.395–9.162, *p* ≤ 0.001). This is probably because increased knowledge of the importance of the vaccination and how it protects one, coupled with the consequences of HBV infection, always pushes one to get vaccinated. Similarly, another study conducted in Cameroon among surgical residents established that the majority (98.0) of the respondents had a good knowledge of risk factors for HBV infection. Although 98.0% were aware of the HBV vaccine, and 89.8% knew that they were at high risk of infection, only 24.5% had received a full course of at least three doses of the vaccine [17]. On the contrary, an institutional-based study conducted in Ethiopia [18] among 370 respondents found that 27.6% of the respondents wrongly responded that one or more doses of HB vaccination are sufficient to be fully immunized for an adult. This is similar to the findings in a study [14] among 288 primary health care workers in Nigeria who found that knowledge of the vaccine increased with an increase in the vaccination dose.

This study found that 77.3% of the respondents said one can get hepatitis B infection through needle stick injury. This is probably because HBV infection is a blood born that can be transmitted through body fluid in which blood is a medium. This is in line with a hospital-based study [19] among 3132 HCWs in Nigeria. It was found that 64.7% of administrative staff did not agree that hepatitis B can be transmitted by needle pricks; a high level of knowledge was registered among all categories of HCWs as 70% acknowledged that blood transfusion can transmit HBV and 3% believed that HBV can be transmitted through handshaking with an infected person, while 16.3% did not know any mode of HBV transmission.

In this study, 76.6% of the respondents said that a hepatitis B infected person may be asymptomatic for a long. This is probably because chronic hepatitis might not manifest as soon as other infections because of the long incubation period. These findings are similar to those from a study conducted in Cameroon among 128 respondents [20] who found that 89.07% of participants were not aware that chronic HBV infection is often asymptomatic. Other studies that had similar findings include the one conducted in Nigeria [21] in which 382 HCWs participated. It was established that 96% were aware of HBV symptoms.

In this study, 46.8% of the respondents were willing to receive HBV vaccinations once offered by chance. Willingness to receive the vaccines was significantly associated with the uptake of hepatitis B vaccination. Respondents who disagreed that they were willing to receive HBV vaccination once offered a chance were less likely to get HBV vaccination compared to those who were willing to receive HBV vaccination once offered a chance (Adjusted OR = 0.698, 95% CI 0.478–0.944, *p* ≤ 0.010), while respondents who agreed that they were willing to receive HBV vaccination once offered chance were less likely to get HBV vaccination compared to those who were not sure about receiving HBV vaccination once offered chance (Adjusted OR = 9.392, 95% CI 6.016–12.165, *p* ≤ 0.004). This is probably because the HCWs were more informed about the risk of HBV infection especially among the health workers who by the nature of their occupation make them more exposed. This concurs with study a conducted in Nigeria among 209 respondents [16]. It was found that all HCWs who perceived themselves at high risk of contracting HBV were willing to receive the vaccine compared to those with low perception risk. Respondent's willingness to receive the vaccination was observed to be influenced by perception level. This similarity in the findings could be due to the same study design that both studies utilized and similar study settings.

In this study, the majority of the respondents who had not completed vaccination (63.0%) were because they were busy, 53.9% forgot about it, 52.6% were waiting for the next dose, and 36.4% felt they were protected. This is probably because the vaccination is not offered at the facility where they work and it is not for free. This is supported by findings from a study conducted in Mangalore [22] among 297 respondents who reported that the majority of the HCWs did not receive vaccination due to various reasons as 28.5% reported forgetting, 66.9% stated lack of time, and 14.7% reported lack of medical benefits and other reasons. Similarly, a study [23] was conducted in Kenya among 266 respondents. It was also found that the reasons for poor vaccination uptake included lack of knowledge of the need for vaccination, unawareness of the procedures or availability of the vaccines, and concerns about side effects.

### 4.3. Health Facility Factors Associated with Hepatitis B Vaccination Uptake

This study found that 51.7% of the respondents said they cannot afford the HBV vaccines and 55.2% of the respondents who were not vaccinated were because they could not afford HBV vaccines. This is because the vaccine is not given for free by the government to the health workers; they have to pay out of pocket. This is consistent with findings from a study conducted in Ethiopia among 423 respondents [9]. It was revealed that none of the health care institutions had made the HB vaccine available, and among the vaccinated respondents, 10 (15.2%) had to pay to get vaccinated and the cost of the vaccine was relatively very high as reported by 137 (33.4%) of the respondents, and 119 (29%) rated it as being expensive. Another study [13] reported that unavailability of vaccines at the health facility, feeling protected with the first or second dose they received, and forgetting to go for other doses were some of the reasons given by the respondents for not being vaccinated.

Availability of HBV vaccines at the hospital was significantly associated with the uptake of hepatitis B vaccination. This is probably because there are no free vaccines supplied by the government to health facilities. These findings are in agreement with those from a study [24] among health care professionals in Ethiopia. It was established that the unavailability of the hepatitis B vaccine decreased the odds of vaccination by 75%. This implies that all the hospitals through the Ministry of Health should be provided with HBV vaccines to service the population.

This study found that 40.3% of the respondents said there were available guidelines followed by all health workers in this facility. Available guidelines followed by all health workers in this facility were significantly associated with uptake of hepatitis B vaccination; respondents who reported the availability of guidelines followed by health workers in the facility were 6.228 times more likely to get HBV vaccination compared to those who said there were no guidelines followed by health workers in the health facility. This is because of the lack of approved guidelines by the MoH in the country. This is consistent with a study that revealed that the availability of hepatitis B policy in the health facility increased the uptake level of the vaccination.

This study found that knowledge of the place of vaccination was significantly associated with uptake of hepatitis B vaccination; respondents who had knowledge of where to get HBV vaccination from were 7.042 times more likely to get HBV vaccination than those who had no knowledge of where to get HBV vaccinations from. This is in line with a study [5] where 98 (65.3%) of the study respondents reported that they had not been offered the vaccine; 70 (46.7%) observed standard precautions to ensure infection prevention and 60 (41.3%) blamed a low level of awareness regarding the availability of the hepatitis B vaccine.

## 5. Conclusions

The uptake of hepatitis B vaccination among health workers at Juba Teaching Hospital was low with only 44.2% implying that HCWs in South Sudan are at greater risk of acquiring hepatitis B infection.

Being married, lack of time, having knowledge about hepatitis B vaccination, and unprotected sexual intercourse are individual factors associated with hepatitis B vaccination.

The availability of the vaccine, the cost, availability of guidelines, and knowledge of where to get HBV vaccination from were the health facility-related factors associated with hepatitis B vaccination uptake.

### 5.1. Recommendations

The government of South Sudan through MoH should ensure that the HBV vaccination policy is adopted and implemented by all hospitals.

All health care workers should be encouraged and supported to embrace the uptake of hepatitis B vaccination.

The government of South Sudan through MoH should first track universal and free vaccination of all HCWs against hepatitis B as recommended by WHO.

MoH of South Sudan should provide the necessary resources to health facilities such as vaccines in the fight against hepatitis B at no cost or at a cost that most health workers can afford.

There is a need to create awareness through training targeted behaviour change communication so that HCWs can recognize the significance of completing the HBV vaccination dose.

## Figures and Tables

**Table 1:** Bivariate analysis of individual factors associated with hepatitis B vaccination.

Variables	Hepatitis B vaccination status		*χ* ^2^	*p* value
	Yes (%)	No (%)		
Hepatitis B infection can be prevented by vaccination				
Yes	63 (73.3%)	59 (86.8%)	4.210	0.031^*∗*^
No	23 (26.7%)	9 (13.2%)		
One can get hepatitis B infection through needle stick injury				
Yes	64 (74.4%)	55 (80.9%)	0.903	0.225
No	22 (25.6%)	13 (19.1%)		
Hepatitis B virus can be found in semen or vaginal fluids of the infected person				
Yes	73 (84.9%)	51 (75.0%)	2.365	0.092
No	13 (15.1%)	17 (25.0%)		
Hepatitis B infected person may be asymptomatic for a long time				
Yes	66 (76.7%)	52 (76.5%)	0.002	0.559
No	20 (23.3%)	16 (23.5%)		
Every person infected by hepatitis can develop acute hepatitis immediately				
Yes	54 (62.8%)	48 (70.6%)	1.032	0.199
No	32 (37.2%)	20 (29.4%)		
Hepatitis B is 10 times more infectious than HIV				
Yes	63 (73.3%)	51 (75.0%)	0.060	0.477
No	23 (26.7%)	17 (25.0%)		
Hepatitis B virus mainly affects the liver				
Yes	65 (75.6%)	53 (77.9%)	0.118	0.441
No	21 (24.4%)	15 (22.1%)		
One can get HBV through unprotected sexual intercourse				
Yes	62 (72.1%)	40 (58.8%)	22.990	0.006^*∗*^
No	24 (27.9%)	28 (41.2%)		
I am aware of where to get hepatitis B vaccination				
Disagree	57 (66.3%)	43 (63.2%)	4.155	0.011^*∗*^
Agree	29 (33.7%)	25 (36.8%)		
I am aware that receiving a full dose of HBV prevents one from HBV infection				
Disagree	16 (18.6%)	15 (22.1%)	2.610	0.271
Agree	38 (44.2%)	36 (52.9%)		
Neutral	32 (37.2%)	17 (25.0%)		
Reuse of used syringes infects one with HBV				
Disagree	11 (12.8%)	13 (19.1%)	2.871	0.238
Agree	57 (66.3%)	36 (52.9%)		
Neutral	18 (20.9%)	19 (27.9%)		
I am aware and I know some of the HBV symptoms				
Disagree	17 (19.8%)	10 (14.7%)	0.840	0.657
Agree	32 (37.2%)	29 (42.6%)		
Neutral	37 (43.0%)	29 (42.6%)		
I am aware that HBV kills faster than HIV				
Disagree	13 (15.1%)	6 (8.8%)	1.883	0.390
Agree	38 (44.2%)	36 (52.9%)		
Neutral	35 (40.7%)	26 (38.2%)		
I am aware that one is supposed to first screen for HBV before vaccination is done				
Disagree	18 (20.9%)	16 (23.5%)	1.839	0.399
Agree	42 (48.8%)	38 (55.9%)		
Neutral	26 (30.2%)	14 (20.6%)		
Poor management of infectious medical waste predisposes me to HBV infection				
Disagree	5 (5.8%)	12 (17.6%)	7.035	0.030^*∗*^
Agree	55 (64.0%)	32 (47.1%)		
Neutral	26 (30.2%)	24 (35.3%)		
All HCWs are at high risk of HBV infection				
Disagree	18 (20.9%)	14 (20.6%)	6.054	0.048^*∗*^
Agree	43 (50.0%)	45 (66.2%)		
Neutral	25 (29.1%)	9 (13.2%)		
Disagree				
Agree				
Neutral				
I am at very high risk of acquiring hepatitis B virus				
Disagree	39 (45.3%)	34 (50.0%)	0.329	0.340
Agree	47 (54.7%)	34 (50.0%)		
My work puts me at high risk of HBV infection				
Disagree	56 (65.1%)	46 (67.6%)	0.109	0.438
Agree	30 (34.9%)	22 (32.4%)		
I am at low risk of acquiring HBV				
Disagree	30 (34.9%)	49 (72.1%)	21.006	0.000^*∗*^
Agree	56 (65.1%)	19 (27.9%)		
Neutral				
I am at no risk of HBV exposure				
Disagree	47 (54.7%)	42 (61.8%)	0.788	0.235
Agree	39 (45.3%)	26 (38.2%)		
I am susceptible to HBV infection				
Disagree	60 (69.8%)	34 (50.0%)	7.014	0.030^*∗*^
Agree	26 (30.2%)	33 (48.5%)		
Neutral	0 (0.0%)	1 (1.5%)		
Hepatitis B infection is very severe				
Disagree	24 (27.9%)	12 (17.6%)	5.886	0.053
Agree	54 (62.8%)	41 (60.3%)		
Neutral	8 (9.3%)	15 (22.1%)		
HBV vaccine is of no benefit				
Disagree	61 (70.9%)	48 (70.6%)	0.002	0.551
Agree	25 (29.1%)	20 (29.4%)		
Neutral				
I am willing to receive HBV vaccination ones offered a chance				
Disagree	34 (39.5%)	19 (27.9%)	14.109	0.028^*∗*^
Agree	34 (39.5%)	38 (55.9%)		
Neutral	18 (20.9%)	11 (16.2%)		
I am willing to pay for HBV vaccination				
Disagree	25 (29.1%)	18 (26.5%)	0.162	0.922
Agree	38 (44.2%)	32 (47.1%)		
Neutral	23 (26.7%)	18 (26.5%)		
To receive health education on HBV vaccination				
Disagree	26 (30.2%)	22 (32.4%)	0.970	0.616
Agree	31 (36.0%)	28 (41.2%)		
Neutral	29 (33.7%)	18 (26.5%)		
To manage hospital infectious waste properly				
Disagree	37 (43.0%)	20 (29.4%)	9.105	0.011^*∗*^
Agree	45 (52.3%)	35 (51.5%)		
Neutral	4 (4.7%)	13 (19.1%)		
To secure time to go for HBV vaccination				
Disagree	43 (50.0%)	39 (57.4%)	18.545	0.000^*∗*^
Agree	39 (45.3%)	13 (19.1%)		
Neutral	4 (4.7%)	16 (23.5%)		

^*∗*^Values that are statistically significant at bivariate analysis level.

**Table 2:** Bivariate analysis of health facility factors associated with hepatitis B vaccination.

Variables	Hepatitis B vaccination status		*χ* ^2^	*p* value
	Yes (%)	No (%)		
Health facility have HBV vaccines				
Yes	28 (41.2%)	30 (34.9%)	9.640	0.023^*∗*^
No	40 (58.8%)	56 (65.1%)		
Affordability of the vaccine				
Yes	14 (50.0%)	14 (46.7%)	15.064	0.004^*∗*^
No	14 (50.0%)	16 (53.3%)		
Health facility offers free vaccination services to health care workers				
Yes	18 (26.5%)	16 (18.6%)	1.366	0.165
No	50 (73.5%)	70 (81.4%)		
Distance from the place of residence from the vaccination point				
<1 km	17 (25.0%)	17 (19.8%)	4.612	0.203
2-3 km	17 (25.0%)	26 (30.2%)		
3-4 km	19 (27.9%)	33 (38.4%)		
>5 km	15 (22.1%)	10 (11.6%)		
Distance hinder accessing the HBV vaccination services				
Yes	33 (48.5%)	35 (40.7%)	0.945	0.209
No	35 (51.5%)	51 (59.3%)		
Attitude of vaccinators				
Very good	14 (20.6%)	18 (20.9%)	2.218	0.528
Good	25 (36.8%)	26 (30.2%)		
Poor	18 (26.5%)	20 (23.3%)		
Very poor	11 (16.2%)	22 (25.6%)		
Ever fail to receive HBV vaccination because health care workers were not present				
Yes	42 (61.8%)	54 (62.8%)	0.017	0.514
No	26 (38.2%)	32 (37.2%)		
Time on average do you spend at the facility before being attended to				
<15 minutes	12 (17.6%)	13 (15.1%)	2.711	0.258
15–30 minutes	34 (50.0%)	34 (39.5%)		
>1 hours	22 (32.4%)	39 (45.3%)		
The facility has personal protective equipment				
Yes	37 (54.4%)	48 (55.8%)	0.030	0.496
No	31 (45.6%)	38 (44.2%)		
Frequency of use				
Daily, so long as I am on duty	7 (17.9%)	12 (24.0%)	15.333	0.000^*∗*^
Once in a while	24 (61.5%)	11 (22.0%)		
I do not wear them	8 (20.5%)	27 (54.0%)		
Reasons for not wearing the personal protective equipment				
Discomfort caused by the PPEs	7 (24.1%)	6 (16.7%)	3.603	0.308
I regard them unsafe	9 (31.0%)	19 (52.8%)		
Difficult to use	9 (31.0%)	9 (25.0%)		
Waists time	4 (13.8%)	2 (5.6%)		
Received any training on the use of personal protective equipment				
Yes	25 (36.8%)	36 (41.9%)	0.412	0.317
No	43 (63.2%)	50 (58.1%)		
Received any health education on the importance of HBV vaccination	33 (48.5%)	48 (55.8%)		
Yes	34 (50.0%)	38 (44.2%)	1.922	0.382
No	1 (1.5%)	0 (0.0%)		
IEC materials are available at this facility				
Posters	27 (39.7%)	33 (38.4%)	9.662	0.022^*∗*^
Brochures	31 (45.6%)	24 (27.9%)		
News papers	5 (7.4%)	20 (23.3%)		
Art models	5 (7.4%)	9 (10.5%)		
Availability of HBV vaccination guidelines at this health facility				
Yes	29 (42.6%)	53 (61.6%)	5.496	0.014^*∗*^
No	39 (57.4%)	33 (38.4%)		
Available guidelines followed by all health workers in this facility				
Yes	23 (33.8%)	39 (45.3%)	2.097	0.100
No	45 (66.2%)	47 (54.7%)		
Availability of policy that guides for the new health workers to be screened against HBV before commencing their duties				
Yes	28 (41.2%)	39 (45.3%)	0.269	0.362
No	40 (58.8%)	47 (54.7%)		
Availability of treatment given to those who are screened positive with HBV				
Yes	24 (35.3%)	32 (37.2%)	0.060	0.470
No	44 (64.7%)	54 (62.8%)		
Knowledge of where to get HBV vaccination from				
Yes	34 (50.0%)	45 (52.3%)	10.082	0.040^*∗*^
No	34 (50.0%)	41 (47.7%)		
Privacy observed at the point of vaccination				
Yes	48 (70.6%)	52 (60.5%)	1.709	0.128
No	20 (29.4%)	34 (39.5%)		
Frequency of conducting vaccination				
Daily	10 (14.7%)	15 (17.4%)	1.364	0.506
Once a week	28 (41.2%)	41 (47.7%)		
Once a month	30 (44.1%)	30 (34.9%)		
Comfortable receiving vaccination from that point				
Yes	28 (41.2%)	33 (38.4%)	0.125	0.425
No	40 (58.8%)	53 (61.6%)		
Reasons for not being comfortable receiving vaccination from that point				
No privacy observed	0 (0.0%)	6 (11.3%)	9.165	0.057
Unfriendly vaccinators	12 (30.0%)	22 (41.5%)		
High cost of vaccination	10 (25.0%)	10 (18.9%)		
Regular absence of vaccinators	17 (42.5%)	12 (22.6%)		
Lack of vaccine	1 (2.5%)	3 (5.7%)		

^*∗*^Values that are statistically significant at bivariate analysis level.

**Table 3:** Multivariate analysis of factors associated with hepatitis B vaccination.

Variables	AOR (95% CI)	*p* value
Marital status		
Married	8.942 (4.313–14.849)	0.008
Not married	1.0	
Hepatitis B infection can be prevented by vaccination		
Yes	4.192 (2.395–9.162)	0.000
No	1.0	
One can get HBV through unprotected sexual intercourse		
Yes	2.135 (1.159–5.371)	0.001
No	1.0	
I am aware of where to get hepatitis B vaccination		
Disagree	0.177 (0.081–0.475)	0.000
Agree	1.0	
I am willing to receive HBV vaccination once offered chance		
Disagree	0.698 (0.478–0.944)	0.010
Agree	9.392 (6.016–12.165)	0.004
Neutral	1.0	
Health facility have HBV vaccines		
Yes	3.258 (1.418–6.497)	0.027
No	1.0	
Available guidelines followed by all health workers in this facility		
Yes	6.228 (3.151–8.902)	0.006
No	1.0	
Knowledge of where to get HBV vaccination from		
Yes	7.042 (4.482–11.196)	0.000
No	1.0	

## Data Availability

The data sets for this study are available and shall be shared upon request.

## Abbreviations

AOR: Adjusted odds ratio
CDC: Centre for disease control
HCWs: Health care workers
CHB: Chronic hepatitis B
CI: Confidence interval
DNA: Deoxyribonucleic acid
GHSS: Global health sector strategy
HBsAg: Hepatitis B surface antigen
HBV: Hepatitis B virus
HCC: Hepatocellular carcinoma
MoH: Ministry of Health
REC: Research and Ethics Committee
WHO: World Health Organization.
